# Development of Gambling Behaviour and Its Relationship with Perceived Social Support: A Longitudinal Study of Young Adult Male Gamblers

**DOI:** 10.1007/s10899-023-10200-7

**Published:** 2023-04-14

**Authors:** Andreas M. Bickl, Ludwig Kraus, Johanna K. Loy, Peter Kriwy, Pawel Sleczka, Larissa Schwarzkopf

**Affiliations:** 1 IFT Institut Für Therapieforschung, Munich, Germany; 2 Institute of Psychology, ELTE, Eötvös Loránd University, Budapest, Hungary; 3 Department of Public Health Sciences, Centre for Social Research on Alcohol and Drugs, Stockholm University, Stockholm, Sweden; 4 Institute of Sociology, Chemnitz University of Technology, Chemnitz, Germany; 5 German University of Health and Sport, Ismaning, Germany; 6 Department of Psychiatry and Psychotherapy, Klinikum der Universität München, Munich, Germany

**Keywords:** Gambling disorder, Social support, Longitudinal study, Young adults

## Abstract

Young adult men who gamble frequently face an elevated risk of developing gambling-related problems. So far, little is known about how changing levels of perceived social support interact with the course of gambling behaviour and gambling-related problems in this population. Using data from a prospective single-arm cohort study (Munich Leisure Time Study), we applied hierarchical linear models to investigate the longitudinal association of changes in perceived emotional and social support (hereafter PESS; operationalized as ENRICHD Social Support Instrument score) with gambling intensity, gambling frequency, and fulfilled criteria for gambling disorder. Pooling data from three time points (baseline, 12-month and 24-month follow-ups) to assess two 1-year intervals, these models disentangle the associations of (a) “level of PESS” (cross-sectional, between participants) and (b) “changes in individual PESS” (longitudinally, within-participants). Among the 169 study participants, higher levels of PESS were associated with fewer gambling-related problems (− 0.12 criteria met; *p* = 0.014). Furthermore, increasing individual PESS was associated with lower gambling frequency (− 0.25 gambling days; *p* = 0.060) and intensity (− 0.11 gambling hours; *p* = 0.006), and fewer gambling-related problems (− 0.19 problems; *p* < 0.001). The results suggest a mitigating influence of PESS on gambling behaviour and gambling-related problems. Increasing individual PESS appears more decisive for this pathway than high initial levels of PESS. Treatment and prevention strategies that activate and reinforce beneficial social resources in people with gambling-related problems are recommended and promising.

## Background

The increasing availability of gambling services via everyday technologies has made gambling convenient and accessible to many segments of the population (Sulkunen et al., 2018). This development is of particular importance for young adult men, who have been repeatedly identified as a risk group for the development of gambling-related problems (Banz, 2019; Buth et al., 2022; Shead et al., 2010; Welte et al., 2008). Although most studies do not explicitly distinguish between adolescence and young adulthood, a recent meta-analysis of studies of college students revealed that approximately 10% of students met the criteria for problem gambling and another 6% met the criteria for pathological gambling. Furthermore, male gender was substantiated as a significant predictor of higher rates of pathological gambling (Nowak, 2018).

Compared to during middle and late adulthood, people in the early stages of life are often less resilient, have less impulse and self-control, and behave in a more risk-seeking manner, i.e., they tend to develop increased risk-taking behaviour, which can be accompanied by feelings of omnipotence (Meyer & Bachmann, 2017). Especially in the context of gambling, these tendencies are reinforced by greater needs for sensation seeking (Riley et al., 2021), illusions of control over the outcome of games (Moore & Ohtsuka, 1999), poor understanding of statistical probability (Delfabbro et al., 2006), and group-oriented behaviour (Zhai et al., 2017). Given this background, the development of effective preventive and treatment strategies that protect young people from gambling-related harms is paramount.

Predictive factors, i.e., risk and protective factors, reflect traits or exposures that supposedly influence the likelihood of problem gambling. So far, a wide range of risk factors associated with problem gambling has been identified in multiple original studies and systematic reviews (Beynon et al., 2020). Focussing on problem gambling in young people, these risk factors are—amongst others—male gender, lower socio-economic status, early gambling onset, an extroverted or impulsive personality, maladaptive coping styles, stress, substance use, ADHD symptoms, anxiety, depression, peer influence, poor academic performance, parental substance abuse or problem gambling, inconsistent parental discipline, and family problems (Dowling et al., 2017; Riley et al., 2021). However, previous research emphasized factors that enhance the risk of problem gambling but paid little attention to protective factors that reduce this risk. These factors are often attributable to interpersonal and community level dimensions (Dowling et al., 2017).

Indeed, a stronger emphasis on social factors as important determinants of problem gambling could be a promising approach to youth-based interventions (Donati et al., 2013). Parental (Vachon et al., 2004; Walters, 2021) and peer gambling behaviour (Langhinrichsen-Rohling et al., 2004; Zhai et al., 2017) have been established as risk factors for the development of problem gambling. On the other hand, social support, defined as the provision of psychological and material resources by a social network, has been identified as a protective counteracting factor (Cohen, 2004). Social support can bolster individuals’ capacities to find alternatives to harmful behaviour, provide moral guidance, and facilitate problem recognition. Social support has been proven to act as a buffer during periods of stress and negative life events in smokers (Hershberger et al., 2016), problem drinkers (Pauley & Hesse, 2009) and people with internet addiction (Esen & Gündoğdu, 2010).

These support mechanisms may also work among people with gambling problems, as some evidence indicates that adolescents who gamble perceive their familial and peer social support as poor (Hardoon et al., 2004) and their social support is lower compared to their peers who do not gamble (Weinstock & Petry, 2008). In addition, low baseline social support level was associated with increased severity of gambling, family, and psychiatric problems, and poorer post-treatment outcomes, in a sample of help-seeking pathological gamblers (Petry & Weiss, 2009). Furthermore, a study of Finnish eighth- and ninth graders revealed that social support from parents and school staff helped reduce gambling frequency (Räsänen et al., 2016).

However, most of the evidence relies on cross-sectional data (Dowling et al., 2017; Hardoon et al., 2004; Räsänen et al., 2016; Weinstock & Petry, 2008), refers exclusively to adolescent or other (i.e. older) adult populations (Bilt et al., 2004; Hardoon et al., 2004), or disregards all gambling-related behaviours and other dimensions except problem severity (Edgerton et al., 2015; Weinstock & Petry, 2008). Studies on longitudinal associations between social support and changes in both gambling behaviour and gambling-related problems are largely lacking, especially for the epidemiologically relevant group of young adult men.

To close some of these knowledge gaps, this paper analyses a cohort of young adult male gamblers with elevated risk of developing gambling-related problems. Based on a study period of two years, it (1) depicts categorical changes in perceived emotional and social support (PESS), gambling-related problems, and gambling behaviour over time; (2) indicates the annual change in their respective outcome measures (ENRICHD Social Support Instrument (ESSI) score; number of gambling disorder (GD) diagnostic criteria fulfilled; gambling frequency and intensity); and (3) investigates how changes in PESS interact with changes in gambling behaviour and gambling-related problems.

## Methods

### Study Design and Procedure

Data stem from assessments performed at baseline (T0), 12-month follow-up (T1), and 24-month follow-up (T2) of the Munich Leisure-time Study (MLS), an online-based longitudinal cohort study of young adult male gamblers conducted between 2014 and 2016. The MLS analysed individuals’ information on gambling behaviour, gambling-related problems, attitudes towards gambling, gambling motives, coping strategies, personality traits, and substance use. In addition, PESS, perceived availability of social resources, and the participants’ social environment were assessed. Further details of the study’s design, measures, and methods have been published elsewhere (Sleczka et al., 2016, 2018).

### Participants

The MLS included males aged 18–25 who were recruited using two methods: (1) random sampling of 25,000 men in that age group drawn from the Munich citizens’ registry and (2) sending open invitations via targeted ads for study participation to Facebook users with gambling listed as an interest on their profiles in order to gather a convenience sample. First, interested persons were screened for gambling behaviour and gambling-related problems. Persons were invited to participate in the longitudinal study (at T0, T1, and T2) if they met at least one of the following requirements: they (a) fulfilled at least one criterion for gambling disorder (GD) (Stinchfield, 2003); (b) scored positively on the Lie-Bet questionnaire (Johnson et al., 1997); or (c) reported gambling once a week (i.e. they answered “yes” to the open question “Do you participate in gambling activities at least once a week?”);

Of the 2588 individuals from the Munich citizens’ registry who completed the screening, 328 were considered eligible. Of those, 115 responded at T0. Five participants were excluded retrospectively because of missing values or inconsistent responses, leaving 110 participants from the citizens’ registry. Another 105 participants were recruited via Facebook. Of those, 12 were excluded due to suspicion of multiple survey responses (based on IP addresses). Of the remaining 93 participants, 70 were considered eligible. After removing seven participants due to repeated non-varying answers, 63 participants from Facebook remained.

Of the entire sample (i.e., participants from Facebook and citizen’s registry) (n = 173), four participants did not respond to items in the ESSI at T0, T1, or T2 and therefore were excluded. Thus, 169 study participants were included in the analyses. Of those, 118 (69.8%) responded only at T1; 124 (73.4%) responded only at T2; and 112 (66.3%) responded at T0, T1, and T2.

### Materials

#### Gambling-Related Problems and Gambling Behaviour

Gambling-related problems during the 12 months prior to T0, T1, and T2 were assessed based on the DSM-IV-oriented “Stinchfield criteria”, a validated tool to operationalize severity of GD (Stinchfield, 2003). The tool (hereafter: GD criteria questionnaire) was translated into German. To adapt it to fit the DSM-5, items about the eighth criterion referring to illegal activities were removed. Of the remaining 17 yes–no items, two items address nine criteria for GD and one item addresses a single criterion (“withdrawal”) (Table B1). Each criterion was considered fulfilled if at least one of the questions regarding that criterion was answered with “yes”. The sum of the number of criteria met was calculated at each time point (T0, T1, T2), yielding a score ranging from 0 to 9 (hereafter: GD score). The GD score’s internal reliability (Cronbach’s alpha) was α = 0.83 at T0, α = 0.91 at T1, and α = 0.85 at T2. Gambling behaviour during the 12 months prior to each time point consisted of two components: (1) the average number of gambling days per month (hereafter: gambling frequency) and (2) the average hours spent gambling per gambling day (hereafter: gambling intensity).

**Table 1:** Participant demographics, PESS, and gambling characteristics, with comparison of participants with and without ESSI score ≤ 18 at baseline

Variables	Baseline (*n* = 169)^†^		ESSI score > 18 (n = 129)^†^		ESSI score ≤ 18 (n = 38)^†^		Comparison test statistics	Associated probability (*p* value)
*Migration background*								
*n*, (%)	*n* = 169		*n* = 129		*n* = 38		2.13^a^	0.144
Yes	46 (27.2%)		32 (24.8%)		14 (36.8%)			
No	121 (72.8%)		97 (75.2%)		24 (63.2%)			
*Partnership status (Having a partner)*								
*n*, (%)	*n* = 164		*n* = 125		*n* = 37		15.31^a^	0.000
Yes	63 (38.4%)		58 (46.4%)		4 (10.8%)			
No	101 (61.6%)		67 (53.6%)		33 (89.2%)			
*Tobacco use within last 12 months*								
*n*, (%)	*n* = 168		*n* = 129		*n* = 38		− 0.67^a^	0.502
Less than twice a week	114 (67.9%)		89 (69.0%)		24 (63.2%)			
Twice per week or more	54 (32.1%)		40 (31.0%)		14 (36.8%)			
*Alcohol use within last 12 months*								
*n, (%)*	*n* = 168		*n* = 127		*n* = 36		1.27^a^	0.205
Less than twice a week	104 (63.4%)		77 (60.6%)		26 (72.2%)			
Twice per week or more	60 (36.6%)		50 (39.4%)		10 (27.8%)			
*ESSI score*^1^	*n* = 167		*n* = 129		*n* = 38		18.86^b^	0.000
*M*, (*SD*)	21.2 (4.2)		23.1 (2.0)		14.8 (3.3)			
*SCL-90-R*								
*Depression Scale (DEP)²*	*n* = 168		*n* = 129		*n* = 38		− 5.00^b^	0.000
*M*, (*SD*)	0.4 (0.6)		0.3 (0.5)		0.8 (0.9)			
*Anxiety Scale (ANX)³*	*n* = 168		*n* = 129		*n* = 38		− 3.44^b^	0.000
*M*, (*SD*)	0.3 (0.5)		0.2 (0.4)		0.5 (0.7)			
*Gambling characteristics*								
GD score^4^	*n* = 169		*n* = 129		*n* = 38		− 1.21^b^	0.229
*M*, (*SD*)	1.4 (2.0)		1.3 (1.9)		1.7 (2.5)			
Gambling frequency (mean number of gambling days/month)	*n* = 160		*n* = 122		*n* = 37		1.64^b^	0.104
*M*, (*SD*)	7.4 (7.8)		8.0 (8.2)		5.6 (6.2)			
Gambling intensity (mean hours of gambling/gambling day)	*n* = 168		n = 129		n = 38		− 1.66^b^	0.100
*M*, (*SD*)	2.2 (2.4)		2.1 (2.2)		2.8 (3.2)			

*PESS* perceived emotional and social support, *ESSI* ENRICHD Social Support Instrument, *GD* gambling disorder

^†^For some analyses, *n* differed due to missings and is reported separately

^a^Pearson chi-square test for ordinal, nominal variables

^b^Student’s t-test for interval variables

^1^ESSI score (measure of PESS): unweighted sum of all ESSI items

^2^SCL-90-R Depression Scale (DEP): unweighted mean of all items on depression

^3^SCL-90-R Anxiety Scale (ANX): unweighted mean of all items on anxiety

^4^GD score (measure of gambling-related problems): sum of no. of fulfilled DSM-5 criteria for GD based on participant endorsement of criteria (via yes–no question)

*p < 0.1, **p < 0.05, ***p < 0.01

#### Level of Perceived Emotional and Social Support (PESS)

PESS was assessed using a standardized, validated German Version of the ENRICHD Social Support Instrument (ESSI) (Cordes et al., 2009; Kendel et al., 2011). The ESSI measures PESS based on five items (Table B2). Each item is rated on a 5-point Likert scale with higher scores indicating a higher level of PESS. The level of PESS was obtained by calculating the unweighted sum of all items (range: 5 to 25). To differentiate between individuals with low and high PESS, a score of ≤ 3 on 2 or more of the items and cut-off value of ≤ 18 for the total score was applied (Kendel et al., 2011). The ESSI was completed at T0, T1, and T2); change in PESS was calculated as the difference between the ESSI score at a given time point and the ESSI score at the previous time point. The internal consistency of the ESSI (Cronbach’s alpha) was α = 0.92 at T0, α = 0.92 at T1, and α = 0.91 at T2.

**Table 2:** Mixed model outputs: changes in PESS, gambling-related problems, gambling frequency, and gambling intensity in entire sample

	T0			T1			T2			First year change^1^ (T0–T1)		Second year change^1^ (T1–T2)	
	Adjusted Mean	SE	(90% CI)	Adjusted Mean	SE	(90% CI)	Adjusted Mean	SE	(90% CI)				
										Δ between T0–T1	*p* value	Δ between T1–T2	*p* value
ESSI score^2^	21.08	0.31	(20.57–21.59)	20.52	0.35	(19.95–21.10)	20.88	0.36	(20.28–21.48)	− 0.56	0.1487	0.36	0.3531
*Gambling characteristics*													
GD score^3^	1.69	0.31	(1.18–2.20)	1.66	0.31	(1.16–2.16)	2.22	0.40	(1.56–2.89)	− 0.03	0.8945	0.56	0.0509*
Gambling frequency^4^	7.97	0.91	(6.47–9.47)	7.32	0.93	(5.79–8.86)	6.07	0.79	(4.78–7.36)	− 0.65	0.4055	− 1.25	0.0943*
Gambling intensity^5^	2.20	0.16	(1.94–2.46)	1.91	0.18	(1.62–2.20)	1.92	0.17	(1.64–2.20)	− 0.29	0.1640	0.01	0.9615

*PESS* perceived emotional and social support, *ESSI* ENRICHD Social Support Instrument, *GD* gambling disorder

Data are presented as adjusted means, standard error (SE), 90% confidence interval (CI)

^1^*p* values based on Wald test statistics

^2^ESSI score (measure of PESS): unweighted sum of all ESSI items

^3^GD score (measure of gambling-related problems): sum of no. of fulfilled DSM-5 criteria for GD based on participant endorsement of criteria (via yes–no question)

^4^Mean no. of gambling days/month

^5^Mean hours spent gambling/gambling day

*p < 0.1, **p < 0.05, ***p < 0.01

#### Covariates Considered

As having a partner is known to positively affect the uptake of treatment for gambling-related problems (Ingle et al., 2008), partnership status (yes/no) at T0 was included as a covariate in our statistical models. Migration background represents a relevant risk factor for the development of maladaptive and problematic gambling behaviour (Donati et al., 2013; Kastirke et al., 2015). Hence, having a migration background (yes/no) (defined as having migrated to Germany oneself or being born in Germany as a (grand)son of people who had immigrated to Germany) was also included in the models.

Initially we also intended to include the onset of gambling (defined as the age when gambling started), because it plays a crucial role in the development of subsequent gambling-related problems (Jiménez-Murcia et al., 2016). As that covariate seriously hampered model convergence without showing any significant association with the gambling characteristics of interest, we refrained from including it.

Furthermore, psychological problems (i.e., depression and anxiety) and concomitant alcohol- or tobacco use are known to be intertwined with gambling behaviour (Jauregui et al., 2016; McGrath & Barrett, 2009). Therefore, we addressed regular alcohol and tobacco use via self-reported consumption patterns in the last 12 months (0 = less than twice a week/1 = twice per week or more) and psychological problems in form of anxiety (10 items) and depression (13 items) via the corresponding subscales (mean value on a 5-point Likert scale) of the Symptom Checklist-90-revised (Derogatis & Unger, 2010) in a sensitivity analysis.

### Statistical Analysis

The entire sample’s characteristics were analysed using summary statistics (means and standard deviations (SD), or frequencies and percentages) at T0. The socio-demographic and gambling characteristics of participants with low and high PESS (ESSI score > 18 vs. ESSI score ≤ 18) were compared using χ^2^ and t-tests.

To evaluate directional changes in gambling behaviour and the extent of gambling-related problems, the differences between values for GD score, gambling frequency, and gambling intensity observed at consecutive time points (Δ T1–T0; Δ T2–T1) were calculated. Subsequently, we calculated the proportions of individuals who remained stable (difference = 0), improved (difference < 0), or deteriorated (difference > 0).

Mixed-effects regression models were used to estimate covariate-adjusted, continuous within-participant changes in GD score, gambling frequency, and gambling intensity. As these outcome measures are count data with right skewed distributions, we used negative binomial models. Since the negative binomial model for changes in the ESSI score did not converge, a Poisson regression model was implemented instead.

To analyse the impact of PESS on gambling behaviour and gambling-related problems, we applied hierarchical linear models (HLM), which are suited to analysing unbalanced longitudinal datasets and allow the simultaneous inclusion of time-invariant and time-variant covariates. In other words, these models enable disentangling the effects of participant-specific changes in a distinct variable (longitudinal, within-participant change in ESSI score) from effects of that variable on the sample level (cross-sectional, between-participant differences in ESSI score at T0, T1, and T2) (Hedeker, 2004). To do so, our model considered observations to be nested in individuals and operationalized the original independent variable (ESSI score) as mean over time (between-participant differences) and the deviation from the mean over time (within-participant change).

1 $${Outcome}_{ji} = \beta 0+ {\beta }_{Time}(Time{)}_{ji}+ {\beta }_{BS}\left({\overline{ESSI}}_{ i }\right)+ {\beta }_{WS}\left({ESSI}_{ ji}- {\overline{ESSI}}_{i }\right)+ {\beta }_{ji}{X}_{ji}+ {\upsilon }_{0i}+ {\varepsilon }_{ji}$$

The HLM is illustrated in Eq. 1 and contains the following terms: a between-participant ${\beta }_{BS}\left({\overline{ESSI}}_{ i}\right)$ and a within-participant ${\beta }_{WS}\left({ESSI}_{ ji}- {\overline{ESSI}}_{ i}\right)$ component; a term accounting for the influence of the individual on his own repeated observations (${\upsilon }_{0i}$), an intercept ($\beta 0$); linear change over time (${\beta }_{Time}(Time{)}_{ji}$); the covariates mentioned above (${\beta }_{ji}{X}_{ji}$); and an error term (${\varepsilon }_{ji}$). The between-participant component ${\beta }_{BS}\left({\overline{ESSI}}_{ i}\right)$ is defined as the person-specific mean value of the ESSI score over all time points and allows determining the influence of higher levels of PESS itself (via cross-sectional, between-participant comparisons). The within-participant component ${\beta }_{WS}\left({ESSI}_{ ji}- {\overline{ESSI}}_{ i}\right)$ reflects how an individual participant’s PESS at a given time point differs from that individual’s mean PESS over all time points. Thus, the influence of increasing PESS (longitudinal, within-participant change) can be modelled.

To examine the robustness of our results and to test whether participants with low PESS differed from those with high PESS at T0, we repeated our analysis, excluding the 38 participants with low levels of PESS at T0 (sensitivity analysis 1; SA1). Within the second sensitivity analysis we additionally accounted for some aspects of psychological burden by including information on concomitant anxiety or depression and regular consumption of alcohol or tobacco, respectively (sensitivity analysis 2; SA2). All statistical analyses were conducted using Stata/SE 15.1 (Stata Corp LP; College Station, TX, USA). An alpha level of 0.1 was used to account for the small sample size.

### Ethics

This study received ethical approval from the ethics committee of the German Association of Psychology (reference number: LK092014).

## Results

### Sample Description

At T0, 27.2% (n = 46) of the 169 participants had a migration background and 38.4% (n = 63) had a partner (Table 1). Within the past 12 months, 32.1% of the 54 participants (n = 54) reported to have used tobacco at least twice per week, 36.6% of the participants (n = 60) admitted consuming alcohol at least twice per week. The participants’ mean ESSI score was 21.2 (SD = 4.2) and their mean GD score was 1.4 (SD = 2.0). On the SCL-90-R depression scale, they scored 0.4 (SD = 0.6), and on the SCL-90-R anxiety scale, their score was 0.3 (SD = 0.5). They gambled on average, 7.4 (SD = 7.8) days per month with an average of 2.2 (SD = 2.4) hours per gambling day. At T0, 23 respondents (13.6%) fulfilled the diagnostic criteria for GD.

Respondents with low levels of PESS less frequently had a partner, achieved higher scores on depression and anxiety, gambled less frequently but with higher intensity than participants with high levels of PESS (Table 1).

### Changes in Perceived Social Support and Gambling-Related Outcomes During the Observation Period

At T1, PESS had increased in 40.2% (n = 47) of the respondents and decreased in 44.4% (n = 52). In parallel, the GD score had improved in 30.8% (n = 52) but deteriorated in 26.0% (n = 44), gambling frequency was reduced in 46.6% (n = 49) and increased in 39.1% (n = 41), and gambling intensity was lower in 34.9% (n = 37) yet higher in 27.4% (n = 29) (Table 4 in"Appendix").

Compared to T1, at T2, PESS had increased in 41.2% (n = 46) and decreased in 38.4% (n = 43) of the respondents. In parallel, the GD score had improved in 30.6% (n = 38) but deteriorated in 32.3% (n = 40), gambling frequency was reduced in 42.7% (n = 44) but increased in 31.1% (n = 32), and gambling intensity was lower in 25.5% (n = 25) but higher in 31.6% (n = 31) (Table 4 in "Appendix").

In the entire sample, no significant changes were detected in ESSI score or gambling characteristics (i.e. gambling-related problems, gambling frequency, gambling intensity) between T0 and T1, whilst between T1 and T2 we observed a statistically significant increase in GD score (0.56; *p* < 0.051) and a statistically significant reduction in gambling frequency (− 1.25; *p* < 0.094) (Table 2).

### Associations of PESS with Development of Gambling-Related Outcomes

Higher levels of PESS (cross-sectional, between-participants comparison) were associated with a significantly improved GD score after twelve months (− 0.12 criteria; *p* = 0.014) (Table 3). Within-participant PESS was associated with the following statistically significant changes after twelve months: improved GD score (− 0.19 criteria; *p* < 0.001), reduced gambling frequency (− 0.25 days; *p* = 0.060), and decreased gambling intensity (− 0.11 h; *p* = 0.006) (Table 3).

**Table 3:** Estimates for associations of cross-sectional PESS level and longitudinal PESS with gambling-related problems, gambling frequency, and gambling intensity

	GD score^1^	Gambling frequency^2^	Gambling intensity^3^
	Estimate [90% CI]	Estimate [90% CI]	Estimate [90% CI]
ESSI score^4^: between-participants	− 0.12**	− 0.17	− 0.05
	[− 0.21 to − 0.02]	[− 0.11 to 0.46]	[− 0.12 to 0.02]
ESSI score^4^: within-participants	− 0.19***	− 0.25*	− 0.11**
	[− 0.26 to − 0.12]	[− 0.51 to 0.01]	[− 0.19 to − 0.03]

*ESSI* ENRICHD Social Support Instrument, *PESS* perceived emotional and social support, *GD* gambling disorder

Hierarchical linear models (HLM) adjusted for migration background and partnership status

Interpretation: Negative estimates indicate improvement in the respective gambling characteristic. ESSI score between-participants: cross-sectional differences in PESS level between participants. ESSI score within-participants: longitudinal change in PESS within individual participants over time

^1^GD score (measure of gambling-related problems): sum of no. of fulfilled DSM-5 criteria for GD based on participant endorsement of criteria (via yes–no question)

^2^Mean no. of gambling days/month

^3^Mean hours spent gambling/gambling day

^4^ESSI score (measure of PESS): unweighted sum of all ESSI items

*p < 0.10; **p < 0.05; ***p < 0.01

### Sensitivity Analysis

Within the SA1 we observed lower mean GD scores and higher mean gambling frequency at T0, T1, and T2, as well as a slightly lower mean gambling intensity at baseline, compared with the main analysis. In addition, the mean PESS was slightly higher at each time point (Table 6 in "Appendix"). Unlike in the main analysis, there was no statically significant increase of the GD score between T0 and T1 (0.35 criteria; *p* = 0.189) but there was a statistically significant decrease of PESS (− 1.69 units; *p* < 0.001). Furthermore, the reduction in gambling frequency between T1 and T2 was more pronounced (− 1.95 days; *p* < 0.045).

Within the HLM models, the between-subject (cross-sectional) comparisons showed associations between PESS level and GD score were more pronounced (− 0.21 criteria; *p* < 0.001) (Table 7 in "Appendix"). Furthermore, there was a statistically significant negative association between PESS level and gambling frequency (− 0.46 days; *p* = 0.017). The within-participant (i.e., longitudinal change) estimators for gambling frequency and gambling intensity effects became weaker and lost statistical significance in the sensitivity analysis (Table 7 in "Appendix").

Within SA2 only regular tobacco consumption was consistently associated with worse GD score as well as worse gambling frequency and intensity. Anxiety and regular alcohol consumption only affected the GD score detrimentally (Table 9 in "Appendix").

Within the HLM models, the between-subject (cross-sectional) comparisons showed associations between PESS level and GD score lost statistical significance and the within participant (i.e., longitudinal change) estimators were less pronounced (− 0.06 criteria; *p* = 0.062; Table 9 in "Appendix"). Vice versa to the main analyses the association between PESS level and gambling frequency was significant for the between estimator (0.28 days; p = 0.074) but not for the within estimator. Regarding gambling intensity, the associations for the within estimator were slightly less pronounced (− 0.10 h; *p* = 0.038).

## Discussion

Pooling data from two consecutive 12-months intervals, we delineated associations of (changing) PESS with gambling-related problems, gambling frequency, and gambling intensity. The analyses revealed that an increasing within-participant PESS over time is associated with beneficial changes in all three gambling characteristics, whilst higher cross-sectional levels of PESS were only associated with reduced gambling-related problems.

Using the established cut-off of an ESSI score ≤ 18 as an indicator for low PESS (Berkman et al., 2000), the mean PESS was inconspicuous in our entire sample at T0 (mean = 21.2). Only 38 of 167 study participants (22.8%) were categorized as having low PESS at T0, which is consistent with an acknowledged ceiling effect of the ESSI score (Kendel et al., 2011). It is worth noting that these participants reported significantly higher gambling intensity at baseline (mean = 2.8 h per day versus mean = 2.1 h per day among those without low PESS; *p* = 0.100), which supports existing evidence on associations between gambling-related problems and low levels of social support (Hardoon et al., 2004; Petry & Weiss, 2009). In contrast, participants with a low PESS reported lower gambling frequency than those with high PESS at T0 (mean = 5.6 vs. mean = 8.0, respectively; *p* = 0.104). This counterintuitive finding might be partially explained by Raymen and Smith’s (2020) argument that gambling is already culturally embedded and normalized as a legitimate and integral feature of the wider masculine weekend leisure experience, and defines itself as a social arena driven by the allure of the gambling win. As it substantiates that hypothesis, it is notable that various (online) gambling services apparently promote peer approval and social pressure to gamble through structural features in the game, further enhancing social aspects (King et al., 2010). Accordingly, study participants may perceive some gambling days as a consciously chosen leisure activity with peers.

Although most individual participants experienced changes in gambling characteristics and PESS during the study period, these developments were not consistently accompanied by changing sample-level means for GD score and gambling behaviour, as improvements and deteriorations compensated for each other. This conflicts with the findings of Petry and Weiss, who reported increases in the average level of social support over 12 months among treatment-seeking individuals with GD who reported an increasing average level of social support among treatment-seeking individuals with GD during the treatment period (Petry & Weiss, 2009) and might be explained by the fact that our sample reflects an at-risk population that presumably has different characteristics than a sample of treatment-seeking individuals diagnosed with GD.

The insight that the individual perspective might be decisive when interpreting longitudinal courses led to our key finding that both the cross-sectional level of PESS and individual changes in PESS over time may improve gambling characteristics. Indeed, our analysis showed that within-participant improvements in PESS were significantly associated with reductions in gambling intensity, gambling frequency, and extent of gambling-related problems, independent of high levels of PESS. Interestingly, the sensitivity analysis of only participants with high PESS at T0 indicated, on average, more favourable gambling characteristics and both reduced relevance of within-participant PESS changes and increased relevance of PESS level (i.e., cross-sectionally). Therefore, we argue the converse can be assumed: individuals with initially low perceived social support may benefit more from improving their individual perceived social supports than people who already have an established supportive social environment.

Previous cross-sectional studies have already demonstrated an association of greater social support with decreased gambling-related problems (Hardoon et al., 2004; van der Maas, 2016; Weinstock & Petry, 2008). Our study expands on that evidence by emphasizing that changing individual-specific PESS is an even more decisive factor in improving gambling characteristics, particularly for people with lower PESS at baseline. Despite being statistically significant, the effect sizes in our study were small. This was anticipated, considering previous evidence on the impact of social support (Peirce et al., 2000) and the comparatively high initial average PESS levels, and the rather mild forms of gambling behaviour of our study participants. Yet evidence of a directional association of social support with gambling behaviour remains inconclusive, as an initially high level of PESS might fail to positively influence problem gambling or its trajectory (Edgerton et al., 2015) or, in cases with poor-quality support, it might even promote gambling (Kalischuk et al., 2006).

Indeed, social support must be understood as an interactive process. It particularly needs to be kept in mind that our approach cannot differentiate whether (a) improving PESS was followed by improving gambling characteristics or (b) improving gambling characteristics preceded rising levels of PESS. Future (quasi-)experimental study designs could help to disentangle this time sequence and thus shed further light on the reciprocal interaction between changing social support and changes in gambling behaviour and extent of gambling-related problems. In addition, further research identifying qualitative characteristics of perceived positive social support, especially within at-risk populations, is recommended so that preventive services can meet individuals’ needs. Support characteristics can depend on situational elements of life context, individual factors such as personality characteristics or expectations, and interpersonal structures of the relationship between support recipient and support provider (Kienle et al., 2006).

### Limitations and Strengths

In addition to the “sequence-caveat”, the following limitations ought to be considered when interpreting this study’s results. First, our study population reflects a city/state-specific convenience sample of young adult males who were regular gamblers. Hence, generalizing our results to other population groups may not be appropriate. This particularly applies to female gamblers. As women are known to usually mobilize, perceive, and experience more social support than men (Matud et al., 2003), we suppose a different role of (changing) PESS is likely among women. Second, owing to the small sample size, and issues with model convergence, subgroup analyses and comprehensive covariate adjustments were limited. Therefore, results have to interpreted very carefully as we might have missed relevant co-predictors that might mitigate or strengthen the observed associations of PESS with gambling outcomes. We included partnership status (having a partner or not) and migration background because an intimate partner is often considered the main source of emotional support, especially among men (Barbee et al., 1993) and because migration backgrounds can strongly affect the thematization of emotions and feelings, e.g. in context of certain role expectations (Durik et al., 2006). A potential moderating role of other factors (e.g., gambling peers, duration of gambling career) was therefore disregarded. We however addressed morbidity aspects within SA 2. As PESS level respectively change remained significant predictors within this model, the results indicate that social support is a robust predictor for several gambling-related outcomes even so effect sizes might have been mis-estimated. Third, the effect sizes found are low, which was expected to some extent, considering that our analyses rely on an at-risk sample and not a clinical sample of people with GD, where different effect sizes would be conceivable. Furthermore, in a meta-analysis of longitudinal studies, it has already been demonstrated, that associations between the development of problem gambling and (social-) risk factors usually only achieve medium to low effect sizes (Dowling et al., 2017). Fourth, the HLMs are used with the assumption that increasing/decreasing social support affects individuals with both high and low initial PESS levels in the same way, even though it appears fair to assume that individuals with low initial PESS levels might benefit from increases more substantially than individuals with higher initial levels of PESS. This was also supported by our sensitivity analysis. This hypothesis should be tested via stratified subgroup analyses or quantile-regression in larger samples (Koenker & Hallock, 2001).

Nevertheless, our focus on young adult males represents a vital contribution to the development of targeted prevention strategies. Here, the preventive potential of social support within a known risk group provides a suitable starting point. Another strength of our study is that social support and gambling behaviour were both operationalized using standardized and validated instruments, which enables a substantial comparison of our study findings to the existing body of evidence. In addition, unlike previous research, our analyses not only examined gambling-related problems but also gambling frequency and gambling intensity, which may be more sensitive to short term changes. Thus, we were able to draw a more comprehensive picture of the associations between gambling behaviour and perceived social support. Finally, the advanced analytical approach differentiated between longitudinal within-participant changes and cross-sectional level effects (between-participant differences), which supports a sound understanding of how different aspects of PESS (both change over time and level at distinct time points) interact with the changes in gambling characteristics.

## Conclusion and Further Research

The results suggest that strengthening social-emotional resources—regardless of the initial cross-sectional level of PESS—may be a promising strategy for mitigating or even preventing gambling-related problems. Since social support is most effective when it matches situational and environmental needs, it is important to create spaces in which young men are allowed to discuss stress and emotional problems that may underlie their gambling behaviour so that an atmosphere of understanding can be established. For young people in particular, online help forums, which have already been evaluated as helpful among people with substance use disorders (Liu et al., 2020) and gambling-related problems (Wood & Wood, 2009), could serve as a first setting where one may perceive social support, even before treatment. Males, who consider mobilization and acceptance of emotional social support generally more challenging than females (Matud et al., 2003), can particularly benefit from the degree of perceived anonymity in online forums. At the same time, social awareness campaigns directed at close contacts of gamblers could raise awareness of the issue of problem gambling and related negative consequences. Given the knowledge of a substantial, enduring association of male gender and young age with gambling-related problems in Germany, development of subpopulation-targeted preventive and early intervention strategies that systematically incorporate the social support networks of young men is paramount.

## Data Availability

The data from this study are available on request from the first author, AB.

## Appendix

See Tables 4, 5, 6, 7, 8, 9, 10, 11.

**Table 4:** Changes in PESS, gambling-related problems, gambling frequency, and gambling intensity from T0 to T1 and T1 to T2

	T0–T1	T1–T2
	Total sample^†^	Total sample
*ESSI score*^a^		
n	117^†^	112^†^
Deterioration	52 (44.4)	43 (38.4)
No change	18 (15.4)	23 (20.5)
Improvement	47 (40.2)	46 (41.2)
*GD score*^b^		
n	169^†^	124^†^
Deterioration	44 (26.0)	40 (32.3)
No change	73 (43.2)	46 (37.1)
Improvement	52 (30.8)	38 (30.6)
*Gambling frequency*^c^		
n	105^†^	103^†^
Deterioration	41 (39.1)	32 (31.1)
No change	15 (14.3)	27 (26.2)
Improvement	49 (46.6)	44 (42.7)
*Gambling intensity*^d^		
N	106^†^	98^†^
Deterioration	29 (27.4)	31 (31.6)
No change	40 (37.7)	42 (42.9)
Improvement	37 (34.9)	25 (25.5)

*PESS* perceived social and emotional support, *ESSI* ENRICHD Social Support Instrument, *GD* gambling disorder

^†^For some analyses, *n* differed due to missings and is reported separately

^a^ESSI score (measure of PESS): unweighted sum of all ESSI items

^b^GD score (measure of gambling-related problems): sum of no. of fulfilled DSM-5 criteria for GD based on participant endorsement of criteria (via yes–no question)

^c^Mean no. of gambling days/month

^d^Mean hours spent gambling/gambling day

**Table 5:** Unadjusted means for ESSI score and gambling outcome measures at baseline (T0) and follow-ups (T1 = 12 months, T2 = 24 months) for total sample and participants with ESSI score ≤ 18 at T0

Variables	Total sample			ESSI ≤ 18 (n = 38)^†^		
	*n*	M	SD	*n*	M	SD
*ESSI score*^1^						
T0	167	21.19	4.20	38	14.82	3.30
T1	126	20.52	4.23	28	17.96	5.68
T2	111	20.72	4.16	31	18.58	4.42
*GD score*^2^						
T0	167	1.38	2.06	38	1.74	2.53
T1	118	2.37	2.86	38	1.00	2.28
T2	124	2.10	2.54	31	2.26	2.63
*Gambling frequency*^3^						
T0	159	7.40	7.81	37	5.57	6.24
T1	111	6.41	7.24	28	3.82	3.48
T2	119	5.62	7.20	31	5.39	7.22
*Gambling intensity*^4^						
T0	167	2.25	2.43	38	2.82	3.16
T1	107	1.89	1.65	28	1.96	1.67
T2	118	1.95	1.83	30	1.97	1.61

*ESSI* ENRICHD Social Support Instrument, *M* unadjusted mean, *SD* standard deviation

^†^For some analyses, n differed due to missings and is reported separately

^1^ESSI score (measure of PESS): unweighted sum of all ESSI items

^2^GD score (measure of gambling-related problems): sum of no. of fulfilled DSM-5 criteria for GD based on participant endorsement of criteria (via yes–no question)

^3^Mean no. of gambling days per month

^4^Mean hours spent gambling/gambling day

**Table 6:** Mixed model outputs from sensitivity analysis 1 (excluding participants with ESSI score ≤ 18 at T0 (n = 38)): changes in PESS, gambling-related problems, gambling frequency, and gambling intensity

	T0			T1			T2			First year change^1^ (T0–T1)		Second year change^1^ (T1–T2)	
	Adjusted Mean	SE	(90% CI)	Adjusted Mean	SE	(90% CI)	Adjusted Mean	SE	(90% CI)	Δ between T0–T1	*p* value	Δ between T1–T2	*p* value
ESSI score^2^	23.01	0.17	(22.72–23.29)	21.32	0.34	(20.76–21.88)	21.62	0.42	(20.93–22.31)	− 1.69	0.0000***	0.30	0.4767
*Gambling characteristics*													
GD score^3^	1.54	0.31	(1.02–2.05)	1.89	0.37	(1.29–2.50)	1.96	0.40	(1.30–2.62)	0.35	0.1887	0.07	0.8247
Gambling frequency^4^	8.76	1.11	(6.93–10.59)	8.13	1.17	(6.20–10.06)	6.18	0.92	(4.67–7.70)	− 0.63	0.5221	− 1.95	0.0449**
Gambling intensity^5^	2.05	0.16	(1.78–2.32)	1.91	0.20	(1.58–2.25)	1.92	0.19	(1.60–2.23)	− 0.14	0.5722	0.01	0.9775

*PESS* perceived emotional and social support, *ESSI* ENRICHD Social Support Instrument, *GD* gambling disorder

Data are presented as adjusted means, standard error (SE), 90% confidence interval (CI)

^1^*p* values based on Wald test statistics

^2^ESSI score (measure of PESS): unweighted sum of all ESSI items

^3^GD score (measure of gambling-related problems): sum of no. of fulfilled DSM-5 criteria for GD based on participant endorsement of criteria (via yes–no question)

^4^Mean no. of gambling days/month

^5^Mean hours spent gambling/gambling day

**p* < 0.10; ***p* < 0.05; ****p* < 0.01

**Table 7:** Estimates for associations of cross-sectional PESS level and longitudinal PESS with gambling-related problems, gambling frequency, and gambling intensity from sensitivity analysis 1 (excluding participants with ESSI score ≤ 18 at T0 (n = 38))

Outcome	GD score^1^	Gambling frequency^2^	Gambling intensity^3^
	Estimator [90% CI]	Estimator [90% CI]	Estimator [90% CI]
ESSI score^4^: between-subjects	− 0.21***	− 0.46**	− 0.03
	[− 0.30 to–0.12]	[− 0.83 to–0.08]	[− 0.14 to 0.07]
ESSI score^4^: within-subjects	− 0.25***	0.00	− 0.04
	[− 0.40 to − 0.10]	[− 0.50 to 0.50]	[− 0.15 to 0.07]

*ESSI* ENRICHD Social Support Instrument, *GD* gambling disorder, *PESS* perceived emotional and social support

Hierarchical linear models (HLM) adjusted for migration background and partnership status; model for gambling frequency did not converge

Interpretation: Negative estimates indicate improvement in the respective outcome parameter. ESSI between-participants: cross-sectional difference in PESS between subjects. ESSI within-participants: longitudinal change in PESS in individuals over time

^1^GD score (measure of gambling-related problems): sum of no. of fulfilled DSM-5 criteria for GD based on participant endorsement of criteria (via yes–no question)

^2^Mean no. gambling days/month

^3^Mean hours spent gambling/gambling day

^4^ESSI score (measure of PESS): unweighted sum of all ESSI items

**p* < 0.10; ***p* < 0.05; ****p* < 0.01

**Table 8:** Mixed-effects negative-binomial and Poisson regression estimates for associations of time, partnership status, and migration background with PESS, gambling-related problems, gambling frequency, and gambling intensity in entire sample and in participants with ESSI score ≤ 18 at baseline (n = 38)

Covariates	ESSI score^1^ (Poisson)		GD score^2^ (negative binomial)		Gambling frequency^3^ (negative binomial)		Gambling intensity^4^ (negative binomial)	
	Entire sample (IRR)	Reduced sample (IRR)	Entire sample (IRR)	Reduced sample (IRR)	Entire sample (IRR)	Reduced sample (IRR)	Entire sample (IRR)	Reduced sample (IRR)
*Time*								
T0	REF	REF	REF	REF	REF	REF	REF	REF
T1	0.97	0.93***	0.98	1.23	0.92	0.93	0.87	0.94
	(0.02)	(0.02)	(0.14)	(0.19)	(0.09)	(0.11)	(0.09)	(0.11)
T2	0.99	0.94***	1.31*	1.28	0.76***	0.71***	0.87	0.94
	(0.02)	(0.02)	(0.19)	(0.21)	(0.08)	(0.09)	(0.09)	(0.11)
*Partnership status (having a partner)*								
No	REF	REF	REF	REF	REF	REF	REF	REF
Yes	1.13***	1.07***	0.80	0.81	0.90	0.84	1.00	1.11
	(0.03)	(0.03)	(0.16)	(0.19)	(0.11)	(0.12)	(0.12)	(0.14)
*Migration background*								
No	REF	REF	REF	REF	REF	REF	REF	REF
Yes	0.95	1.01	0.69*	0.61*	0.96	0.88	0.84	0.68**
	(0.03)	(0.03)	(0.15)	(0.16)	(0.13)	(0.14)	(0.11)	(0.10)
*Observations*	395	297	448	337	380	282	381	283
*Number of groups*	164	125	164	125	161	123	164	125

*ESSI* ENRICHD Social Support Instrument, *GD* gambling disorder

Standard errors in parentheses; estimators reported as incidence rate ratios (IRR)

^1^ESSI score (measure of PESS): unweighted sum of all ESSI items

^2^GD score (measure of gambling-related problems): sum of no. of fulfilled DSM-5 criteria for GD based on participant endorsement of criteria (via yes–no question)

^3^Mean no. of gambling days per month

^4^Mean hours spent gambling/gambling day

**p* < 0.10; ***p* < 0.05; ****p* < 0.01

**Table 9:** Estimates for associations of covariables, morbidity cross-sectional PESS level and longitudinal PESS with gambling-related problems, gambling frequency, and gambling intensity from sensitivity analysis 2

Covariates	GD score^2^		Gambling frequency^3^		Gambling intensity^4^	
	Main analysis	Morbidity adjusted	Main analysis	Morbidity adjusted	Main analysis	Morbidity adjusted
*ESSI*^1^						
ESSI score: between-subjects	− 0.12**	− 0.05	0.17	0.28*	− 0.05	− 0.03
	(0.05)	(0.05)	(0.15)	(0.15)	(0.04)	(0.04)
ESSI score: within-subjects	− 0.19***	− 0.06*	− 0.25*	0.00	− 0.11***	− 0.10**
	(0.03)	(0.03)	(0.13)	(0.15)	(0.04)	(0.05)
*Time*						
T0	REF	REF	REF	REF	REF	REF
T1	0.86***	0.44**	− 0.78	− 1.70**	− 0.42*	− 0.51*
	(0.21)	(0.18)	(0.80)	(0.81)	(0.24)	(0.26)
T2	0.57***	0.40**	− 1.67**	− 1.79**	− 0.36	− 0.35
	(0.20)	(0.17)	(0.77)	(0.74)	(0.23)	(0.24)
*Partnership status (having a partner)*						
No	REF	REF	REF	REF	REF	REF
Yes	− 0.17	− 0.08	− 1.33	− 1.16	0.25	0.34
	(0.35)	(0.32)	(1.05)	(1.02)	(0.26)	(0.27)
*Migration background*						
No	REF	REF	REF	REF	REF	REF
Yes	− 0.90**	− 0.69**	− 0.52	− 0.51	− 0.38	− 0.25
	(0.36)	(0.33)	(1.08)	(1.06)	(0.27)	(0.28)
*Tobacco Use within last 12 months*						
Less than twice a week		REF		REF		REF
Twice per week or more		0.96***		1.21		0.36
		(0.26)		(0.96)		(0.27)
*Alcohol Use within last 12 months*						
Less than twice a week		REF		REF		REF
Twice per week or more		0.53**		0.39		0.20
		(0.21)		(0.84)		(0.24)
*SCL-90-R*						
*Depression Scale (DEP)*^5^		0.32		0.33		0.38
		(0.20)		(0.83)		(0.25)
*Anxiety Scale (ANX)*^6^		0.66**		1.62		− 0.05
		(0.28)		(1.10)		(0.33)
*Observations*	395	365	379	354	380	355
*Number of groups*	164	162	161	160	164	162

*ESSI* ENRICHD Social Support Instrument, *GD* gambling disorder

Standard errors in parentheses

Interpretation: Negative estimates indicate improvement in the respective outcome parameter. ESSI between-participants: cross-sectional difference in PESS between subjects. ESSI within-participants: longitudinal change in PESS in individuals over time

^1^ESSI score (measure of PESS): unweighted sum of all ESSI items

^2^GD score (measure of gambling-related problems): sum of no. of fulfilled DSM-5 criteria for GD based on participant endorsement of criteria (via yes–no question)

^3^Mean no. of gambling days per month

^4^Mean hours spent gambling/gambling day

^5^SCL-90-R Depression Scale (DEP): unweighted mean of all items on depression

^6^SCL-90-R Anxiety Scale (ANX): unweighted mean of all items on anxiety

**p* < 0.10; ***p* < 0.05; ****p* < 0.01

**Table 10:** Original English language items in questionnaire on gambling-related problems and corresponding gambling disorder (GD) criteria (GD criteria questionnaire)

Gambling disorder (GD) Criteria	Original 17 items measuring DSM-5 criteria for GD (in English)
Needs to gamble with increasing amounts of money in order to achieve the desired excitement	*Have you had periods when you needed to gamble more often in order to obtain the same excitement?*
	*Have you needed to gamble with larger amounts of money or with larger bets in order to obtain the same feeling of excitement?*
Is restless or irritable when attempting to cut down or stop gambling	*Did you feel quite restless or irritable after you tried to cut down or stop gambling?*
Has made repeated unsuccessful efforts to control, cut back, or stop gambling	*Have you tried to cut down or control your gambling several times in the past and found it difficult?*
	*Have you tried to stop gambling several times in the past and been unsuccessful?*
Is often preoccupied with gambling (e.g., having persistent thoughts of reliving past gambling experiences, handicapping or planning the next venture, thinking of ways to get money with which to gamble)	*Have there been periods in the past year when you spent a lot of time thinking about past gambling experiences or thinking about future gambling ventures?*
	*Have you frequently thought about ways of getting money with which to gamble?*
Often gambles when feeling distressed (e.g., helpless, guilty, anxious, depressed)	*Do you feel that you gamble as a way to escape personal problems?*
	*Does gambling seem to relieve uncomfortable emotions, such as anxiety or depression?*
After losing money gambling, often returns another day to get even (“chasing” one’s losses)	*When you lose money on a given day, do you often return soon another day to win back your losses?*
	*When you had a large gambling debt, did you gamble more often in the hopes of winning back your money?*
Lies to conceal the extent of involvement with gambling	*Have you often lied to family members, friends, co-workers or teachers about the extent of your gambling or of your gambling debt?*
	*Have you often hidden or tried to hide your gambling from others (e.g. family members)?*
Has jeopardized or lost a significant relationship, job, or educational or career opportunity because of gambling	*Have you had periods when your gambling caused problems in your relationships with family, friends, co-workers or teachers?*
	*Have you missed work, school or important social or family activities because of gambling?*
Relies on others to provide money to relieve desperate financial situations caused by gambling	*Have you asked people to lend you money because of your financial problems due to gambling?*
	*Have you had others pay your gambling debts for you (i.e. bail you out) when you felt desperate about your financial situation?*

**Table 11:** Original English language items in standardized, validated German version of the ENRICHD Social Support Instrument (ESSI)

Original 5 items in the ESSI (in English)
*Is there someone available to you who you can count on to listen when you need to talk?*
*Is there someone available to give you good advice about a problem?*
*Is there someone available to you who shows you love and affection?*
*Can you count on anyone to provide you with emotional support, such as talking over problems or helping you make difficult decisions?*
*Do you have as much contact as you would like with someone you feel close to, someone you can trust and confide in?*
